# Accurate Risk Assessment of Patients with Pathologic T3aN0M0 Renal Cell Carcinoma

**DOI:** 10.1038/s41598-018-32362-w

**Published:** 2018-09-17

**Authors:** Jong Jin Oh, Jung Keun Lee, Byung Do Song, Hakmin Lee, Sangchul Lee, Seok-Soo Byun, Sang Eun Lee, Sung Kyu Hong

**Affiliations:** 1 Department of Urology, Seoul National University Bundang Hospital, Seongnam-si, South Korea; 2 Department of Urology, Seoul National University College of Medicine, Seoul, South Korea

## Abstract

To develop a more precise risk-stratification system by investigating the prognostic impact of tumor growth within fatty tissues surrounding the kidney and/or renal vein. We conducted a retrospective review of the medical records of 211 patients with a pathologic diagnosis of T3aN0M0RCC among 4,483 renal cell carcinoma (RCC) patients from February 1988 to December 2015 according to the number of T3a pathologies—extrarenal fat invasion (EFI) and/or renal venous invasion (RVI). During a mean follow-up duration of 38.8 months, the patients with both pathologies (EFI + RVI) had lower recurrence free survival (RFS) rate than those with only a single pathology (p = 0.001). Using multivariable Cox regression analysis, the presence of both factors was shown to be an independent predictor of RFS (HR = 1.964, p = 0.032); cancer specific survival rate was not different among patients with EFI and/or RVI. Patients with pathologic T3aN0M0 RCC presenting with both EFI and RVI were at an increased risk of recurrence following nephrectomy. Therefore, pathologic T3a RCC could be sub-divided into those with favorable and unfavorable disease according to presence of EFI and/or RVI pathologies.

## Introduction

Renal cell carcinoma (RCC) is the third most common urologic cancer in the world, making up roughly 3% of all human cancers^1^. Studies have shown that 20~30% RCC recur after surgical treatment, thus highlighting the importance of: i) using prognosticators (eg, pathological staging) when developing a treatment plan and ii) close monitoring during and following treatment^2–5^. The 2002 RCC Tumor-Node-Metastasis (TNM) staging system defined T3 RCC as a tumor that extends to fat, adrenal or major vein. In 2009 however, this system was updated to better differentiate and improve prognosis of T3 tumors^6–8^. In the 2009 version, renal vein invasion was down-staged from T3b to T3a and adrenal invasion was up-staged from T3a to T4 or M1^6^.

In the current staging system, T3a RCC is defined as a tumor with extra-renal fat invasion (EFI) (perinephric or sinus fat invasion) and renal venous invasion (RVI) (renal vein or its segmental muscle containing branch)^9^. The limited data available comparing the prognostic significance of EFI and/or RVI are inconsistent^10,11^. For instance, some studies have suggest a significant impact of RVI on clinical outcome, while others have reported no prognostic value to the presence of tumor thrombus^12^. Other studies show that RCC with both EFI and RVI have higher recurrence rates than those with only EFI or RVI^10^.

Currently, a T3a RCC tumor may have EFI and/or RVI and the prognostic significance of these characteristics is unclear. We hypothesize that T3 tumors with EFI and RVI are more aggressive than those with only EFI or RVI and have designed the current research study to compare the impact of these features in non-metastatic T3a tumors (ie, prognosis, survival).

## Materials and Methods

An approval of this study was obtained from institutional review board approval (B-1712/381-108, and written informed consent was acquired from all patients before the inclusion to the research.

### Patient selection

After receiving institutional review board approval, we reviewed the data of all patients (n = 4,483) who underwent a partial or radical nephrectomy for RCC between February 1988 and December 2015 from two tertiary hospital (Seoul National University Hospital and Seoul National University Bundang Hospital). Among them, we identified 211 patients with T3aN0M0 RCC for this analysis.

### Data collection protocol

Postoperative pathologic tumor stages and grades were determined in accordance with the seventh AJCC TNM staging^13^ and Fuhrman grading system^14^, respectively. Histological subtypes of RCC were assigned in accordance with the WHO classification system^15^. We evaluated the following clinical and pathologic features: age at surgery, gender, body mass index, Eastern Cooperative Oncology Group performance (ECOG) performance status, surgery type (partial nephrectomy [PN] or radical nephrectomy [RN]), tumor location, tumor size, histologic subtype (clear cell, papillary, chromophobe and unclassified), Fuhrman nuclear grade, capsular invasion, presence of necrosis, angiolymphatic invasion, perineural invasion and sarcomatoid differentiation. Additionally, T3a tumors were classified as: i) isolated EFI, isolated RVI, or iii) EFI and RVI.

### Follow up protocol

After nephrectomy, all patients were followed up on according to the SNU protocol. Symptom assessments, routine blood tests, chest x-rays and kidney/bladder Computed tomography (CT) scans were conducted every 3 months for the first 2 years. Following year 2, the same battery of tests were conducted every 6 months for 2 years followed by annual follow ups as necessary.

### Statistical analysis

Comparisons were conducted across the T3a patient classifications (ie, EFI, RVI, and EFI + RVI). Another group analysis was performed according to the number of cause of pT3a (ie, isolated EFI or RVI, and EFI + RVI). Analyses were performed using SPSS for Windows, version 22.0 (SPSS Inc., Chicago,IL, USA). Fisher’s exact and Pearson chi-squared tests were performed to compare clinical and pathological features among groups. Differences in variables with a continuous distribution across dichotomous categories were assessed using the Mann –Whitney U -test. A P value of <0.05 was considered statistically significant.

Recurrence was defined as radiologically verified metastasis or local disease recurrence during the study period. Recurrence-free survival (RFS) was defined as the interval between the primary surgery and the last follow-up visit and the absence of disease recurrence or progression. Cancer-specific survival (CSS) was defined as the interval between the primary surgery and the last follow-up visit or RCC related death. The RFS and CSS in each group were estimated using Kaplan-Meier methods and the log-rank test was applied to compare survival curves. Univariate and Multivariate Cox proportional hazard models were used to verify predictors of RFS and CSS.

## Results

### Baseline characteristics of pT3a RCC patients

The clinical and pathological characteristics of 211 T3aN0M0 RCC patients who underwent nephrectomy are summarized in Table 1. This study included 158 male patients (74.9%) and 53 female patients (25.1%). The median age (IQR) was 60 years old (52–69), mean BMI (IQR) was 23.9 (22.3–26.2) and most patients had ECOG performance status 0–1 (95.3%). Among 211 patients, 26 patients (12.3%) underwent PN. Median tumor size (IQR) was 6.8 centimeter (4.9–9.4), and 81.5% of tumors were classified as clear cell type. EFI was present in 173 patients (82.0%) and RVI was present in 84 (39.8%); 47 patients had EFI and RVI (23.3%). Total of 124 (58.8%) patients had only EFI and 40 (19.0%) patients had only RVI as cause of pT3a, respectively. The comparison of baseline characteristics according to groups were shown in Table 1.

**Table 1:** Clinical and pathological characteristics of the patients with pT3a renal cell carcinoma according to cause of pathologic T3a.

Variables	Total	EFI only	RVI only	EFI + RVI	p-value
No. patients	211	124	40	47	
Median age (year)(IQR)	60.0 (52–69)	58.5 (50.0–69.0)	62.8 (54.0–71.0)	62.7 (56.0–69.0)	0.041
Female (%)	53 (25.1)	30 (24.2)	13 (32.5)	10 (21.3)	0.509
Median body mass index (kg/m^2^)(IQR)	23.9 (22.3–26.2)	24.4 (22.6–26.3)	24.5 (22.1–26.3)	24.0 (21.7–26.2)	0.690
ECOG performance status (%)					0.475
0–1	201 (95.3)	120 (96.8)	38 (95.0)	44 (93.6)	
2–3	10 (4.7)	4 (3.2)	2 (5.0)	3 (6.4)	
Partial nephrectomy (%)	26 (12.3)	22 (17.7)	1 (2.5)	3 (6.3)	0.012
Median pathologic tumor diameter (cm)(IQR)	6.8 (4.9–9.4)	6.5 (4.5–9.5)	7.2 (4.5–9.1)	7.0 (5.0–9.4)	0.600
Histological type (%)					0.489
Clear cell	172 (81.5)	100 (80.6)	34 (85.0)	38 (80.9)	
Papillary	15 (7.1)	10 (8.1)	1 (2.5)	4 (8.5)	
Chromophobe	18 (8.5)	11 (8.9)	3 (7.5)	4 (8.5)	
Others	6 (2.9)	3 (2.4)	2 (5.0)	1 (2.1)	
Fuhrman grade (%)					0.195
1–2	45 (20.3)	31 (25.0)	4 (10.0)	9 (19.1)	
3–4	166 (78.7)	93 (75.0)	36 (90.0)	38 (80.9)	
Positive surgical margin (%)	4 (1.9)	2 (1.6)	1 (2.5)	1 (2.1)	0.939
Capsular invasion (%)	152 (72.0)	102 (82.3)	12 (30.0)	38 (80.9)	<0.001
Necrosis (%)	103 (48.8)	59 (47.6)	18 (45.0)	26 (55.3)	0.541
Angiolymphatic invasion (%)	33 (15.6)	10 (8.1)	9 (22.5)	14 (29.8)	0.001
Perineural invasion (%)	6 (2.8)	3 (2.4)	1 (2.5)	2 (4.3)	0.804
Sarcomatoid differentiaton (%)	26 (12.3)	11 (8.9)	3 (7.5)	12 (25.5)	0.007

IQR; interquartile range, ECOG; Eastern Cooperative Oncology Group, EFI; isolated extracapsular fat invasion, RVI; isolated renal venous invasion.

### Recurrence-free survival

**Figure 1:**
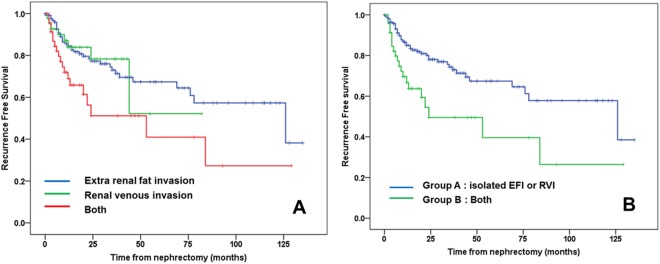
Recurrence free survival of 211 patients with T3aN0M0 renal cell carcinoma patients according to presence of extrarenal fat invasion (EFI) and/or renal venous invasion (RVI). Comparison by Kaplan-meier analysis showed each group (EFI vs. RVI vs. both) (**A**), comparison by the number of pathologis (EFI/RVI) – isolated EFI or RVI vs. both group (**B**).

During a mean follow-up of 38.8 months (median 26.0 months), radiologic recurrence was observed in 42 patients (19.9%). The site of recurrence was recorded as renal fossa (28/42, 66.6%), lymph node (8/42, 19.0%) and lung (4/42. 9.5%). RFS was significantly lower in EFI + RVI group than either EFI only or RVI only groups (Fig. 1A, log rank test = 0.008 or 0.010, respectively). According to the number of parameters leading to T3a classification, which Group B (EFI + RVI) had a significantly lower RFS rate than Group A (EFI only + RVI only) (Fig. 1B, p = 0.002). Seven-year RFS rates were calculated as 57.8% in Group A and 26.4% in Group B.

As shown in Table 2, sex, tumor size, tumor necrosis, perineural invasion, angiolymphatic invasion, sarcomatoid differentiation and having both EFI and RVI (Group B) were significant factors in univariate Cox proportional hazard analysis and predictive of tumor recurrence. Using a multivariate analysis, sex, tumor size, tumor necrosis, and having both EFI and RVI (Group B) were shown to be significant factors.

**Table 2:** Uni- and Multi-variable Cox-regression analyses on recurrence free survival.

Variables	HR (95% CI)	p-value	HR (95%CI)	p value
Univariate analysis			Multivariate analysis	
Age	1.005 (0.984–1.027)	0.650		
Female	1.905 (1.131–3.207)	0.015	2.222 (1.291–3.825)	0.003
Body mass index	0.929 (0.851–1.015)	0.101		
ECOG performance status	2.326 (0.937–6.463)	0.105		
Tumor diameter	1.163 (1.103–1.227)	<0.001	1.107 (1.047–1.170)	<0.001
Surgical methods (RN vs PN)	0.634 (0.273–1.474)	0.290		
Surgical margin positive	0.955 (0.475–1.919)	0.897		
Histologic type (clear cell vs.)	1.272 (0.700–2.313)	0.430		
Fuhrman grade (1–2 vs 3–4)	1.624 (0.859–3.072)	0.136		
Extra-renal fat invasion	5.154 (4.264–6.230)	<0.001		
Renal venous invasion	6.175 (5.050–7.550)	<0.001		
pT3a factor (2 vs 1)	2.254 (1.319–3.850)	0.003	1.961 (1.059–3.632)	0.032
Capsular invasion	1.099 (0.624–1.935)	0.744		
Necrosis	3.100 (2.777–3.461)	<0.001	3.163 (1.760–5.684)	<0.001
Perineural invasion	7.681 (4.091–14.420)	<0.001	1.795 (0.487–6.624)	0.380
Angiolymphatic invasion	6.356 (5.004–8.074)	<0.001	1.923 (0.985–3.753)	0.055
Sarcomatoid differentiation	1.412 (1.305–1.529)	<0.001	1.409 (0.694–2.863)	0.343

HR; hazard ratio, CI; confidence interval, ECOG; Eastern Cooperative Oncology Group, RN; radical nephrectomy, PN; partial nephrectomy.

### Cancer-specific survival

**Figure 2:**
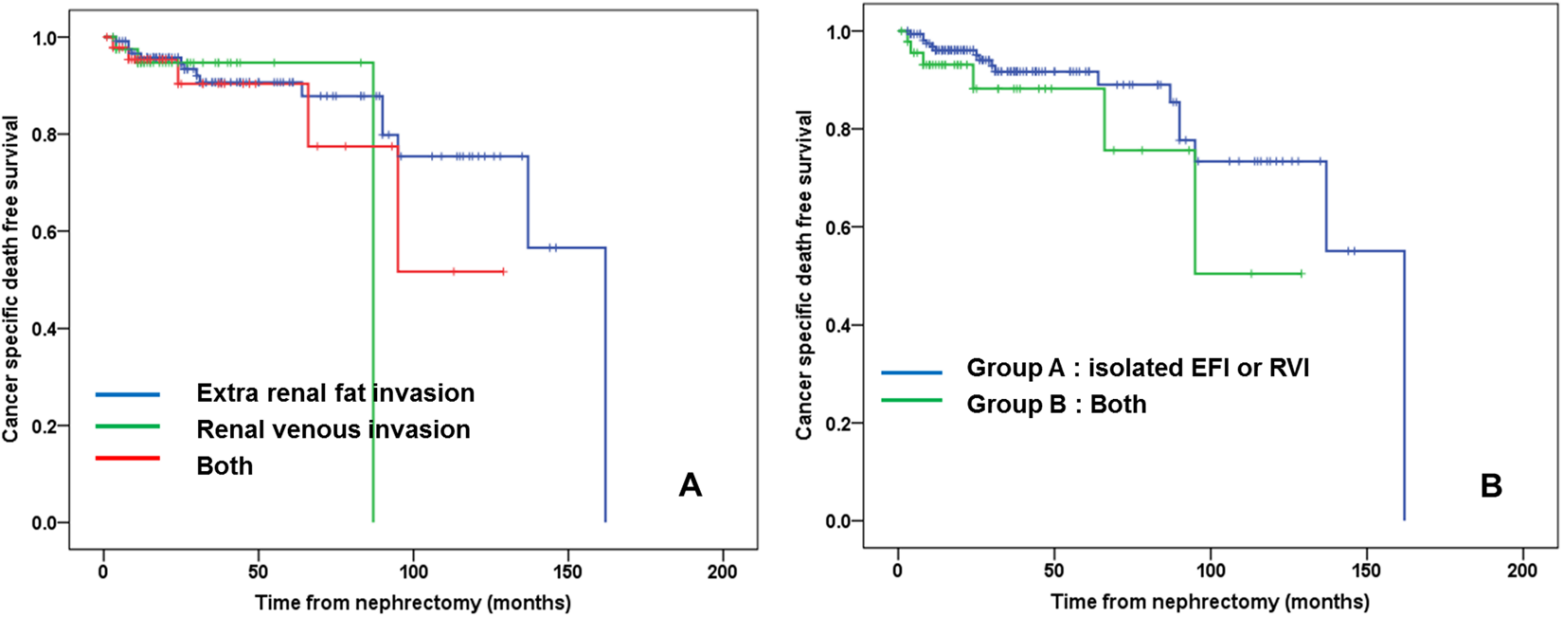
Cancer specific survival of 211 patients with T3aN0M0 renal cell carcinoma patients according to presence of extrarenal fat invasion (EFI) and/or renal venous invasion (RVI). Comparison by Kaplan-meier analysis showed each group (EFI vs. RVI vs. both) (**A**), comparison by the number of pathologis (EFI/RVI) – isolated EFI or RVI vs. both group (**B**).

During follow-up, 23 patients died due to RCC. CSS free survival was lower in the EFI + RVI group than either EFI-only or RVI-only group, however this difference was not statistically significant (Fig. 2, log rank test = 0.625, 0.882, respectively). Using a multivariate Cox proportional hazard analysis, tumor size was shown to be a significant factor in determining CSS among T3a RCC patients who underwent nephrectomy (HR = 1.133, 95% CI: 1.040–1.234, p = 0.004) (Table 3). Another factors (positive surgical margin and presence of tumor necrosis) were significant factors to predict cancer specific survival (HR = 19.864, p < 0.001 and HR = 4.238, p = 0.025, respectively).

**Table 3:** Uni- and Multi-variable Cox-regression analyses on cancer specific survival.

Variables	HR (95% CI)	p-value	HR (95%CI)	p value
Univariate analysis			Multivariate analysis	
Age	0.980(0.945–1.017)	0.290		
Female	1.037 (0.403–2.669)	0.940		
Body mass index	0.973 (0.841–1.125)	0.708		
ECOG performance status	2.660(0.610–11.594)	0.193		
Tumor diameter	1.169 (1.092–1.252)	<0.001	1.133 (1.040–1.234)	0.004
Surgical methods (RN vs PN)	0.368 (0.048–2.748)	0.329		
Surgical margin positive	7.784 (1.758–34.457)	0.007	19.864 (3.803–103.749)	<0.001
Histologic type (clear cell vs.)	1.346 (0.492–3.684)	0.563		
Fuhrman grade (1–2 vs 3–4)	2.216 (0.736–6.672)	0.157		
Extra-renal fat invasion	1.033 (0.232–4.605)	0.966		
Renal vein invasion	1.701 (0.960–4.197)	0.249		
pT3a factor (2 vs 1)	1.901 (0.735–4.918)	0.185	1.907 (1.107–12.301)	0.201
Capsular invasion	0.525 (0.224–1.229)	0.138		
Necrosis	4.760 (1.601–14.148)	0.005	4.238 (1.201–14.957)	0.025
Angiolymphatic invasion	2.166 (0.777–6.035)	0.139		
Sarcomatoid differentiation	3.778 (1.232–11.583)	0.020	1.580 (0.434–5.747)	0.487

HR; hazard ratio, CI; confidence interval, ECOG; Eastern Cooperative Oncology Group, RN; radical nephrectomy, PN; partial nephrectomy.

## Discussion

In this analysis, we reviewed 211 T3aN0M0 RCC patients who underwent surgical treatment, and stratified patients according to the cause of their RCC classification as T3a; the 173 EFI (82.0%) and 84 RVI (39.8%) patients had similar RFS rates during a median follow-up of 26.0 months. However, patients with both EFI and RVI (n = 47, 23.3%) had a significantly lower RFS rate than patients who had only one factor. Despite of no significance to CSS, the patients who had EFI and RVI had more aggressive feature among T3a RCC patients.

Da Costa *et al*.^10^ who reported similar results to ours stratified 46 T3a RCC patients into one of 3 groups: i) EFI only (24 [52.1%]), ii) RVI only (11 [23.9%]), and iii) EFI and RVI (11 [23.9%]). According to their study, patients with both pathologies had poorer RFS rate (roughly 22%) than patients with only one factor. In our study, using larger cohorts, patients with both pathologies had a PFS of 26.4% at 7 years.

According to the current TNM staging system, EFI and RVI can each lead to inclusion in same stage – (T3a) regardless of whether one or both of these pathologies (EFI or RVI) are present. However, several previous studies have shown that patients with EFI and RVI had poorer outcomes than those with only one (EFI or RVI)^10,16–18^. A recent European multi-center study^17^, validated the latest edition of the TNM (2009), evaluating 503 cases of T3a stage RCC and noting significantly lower survival rates in patients with concomitant EFI and RVI vs those with the single pathologies. Margulis *et al*.^18^ confirmed these results by analyzing 419 patients treated for pT3a stage RCC; poorer survival rates were observed in patients with EFI + RVI compared to patients with either pathology alone. Our results indicate a significant difference in RFS among groups A (single pathology) and B (both pathologies), however we did not observe a significant difference among the CSS of the two groups The difference between our study and previous studies is that we excluded patients who had lymph node invasion (LNI)^10,16^. The previous study showed that patients who had EFI and RVI had higher rates of LNI. According to the Da Costa group^10^, patients with both pathologies had much higher rates of LNI than those with a single pathology only. A study by Chen *et al*.^2^ also showed no difference between RVI and/or EFI among T3N0M0 patients. Findings published by Abel *et al*.^19^, showed no difference in CSS according to the presence of RVI with or without EFI. Differences in exclusion criteria showed the somewhat different CSS results between RVI and EFI among our results and previous reported results.

In our results, EFI-only and RVI-only patients had similar RFS and CSS rates. Baccos *et al*.^11^ reported that the CSS was similar in RVI-only and EFI-only patients, Novara *et al*.^17^, in a large multi-institutional study, showed that an RVI-only group had a better prognosis than the fat-invasion group. In contrast, Jung *et al*.^20^ reported that a group of RVI-only patients had lower 2-year and 5-year survival rates than the fat invasion group. Park *et al*.^16^ showed that RVI-only patients had significantly lower RFS and CSS than EFI among T3aN0M0 patients after nephrectomy. Therefore, this issue is still controversial and future prospective studies may be required to confirm the results of these studies.

Another interesting issue in this study was shown in multivaratie analysis to predict RFS and CSS. Regardless of the number of factors of pT3a, tumor diameter and tumor necrosis were significant factors to RFS and CSS, respectively. A previous reports also showed in their study among pT3a RCC patients the importance of tumor size in pT3a, HR to recurrence was 1.125 (p < 0.001)^21^ and HR to CSS was 2.506 (p = 0.011)^2^, it was same result in our study. And another study about tumor necrosis was similar results to our study, they emphasized the significance of tumor necrosis to prognosis in RCC patients^22,23^.

There were several limitations, in our study. Firstly, our study was a retrospective chart review. Secondly, we could not differentiate between EFI into perinephric fat invasion and sinus fat invasion. However, most studies which compare perinephric fat invasion and sinus fat invasion have reported similar results between two groups. Mouracade *et al*.^24^ showed, in their analysis of 143 T3a RCC patients, sinus fat invasion and perinephric fat invasion had similar prognostic values. Poon *et al*.^25^ reported that sinus fat invasion was not a predictor of CSS. Therefore, our inability to stratify patients with EFI into perinephric fat invasion and sinus fat invasion groups could be significant. Despite a relatively long follow up duration (mean of roughly 40 months), only 10% patients died from RCC, therefore CSS analysis was limited by the small sample size. More studies with a longer follow up and more patients from multiple centers could overcome this limitation.

In conclusion, among T3aN0M0 RCC patients, those who had both EFI and RVI had poor PFS rates than patients with only one pathology (ie, EFI or RVI). Therefore, following surgery, T3a patients with EFI and RVI should be more closely followed up than other T3a RCC patients.

## References

[1] DeSantis CE (2014). Cancer treatment and survivorship statistics, 2014. CA Cancer J Clin.

[2] Chen L (2017). Influence of tumor size on oncological outcomes of pathological T3aN0M0 renal cell carcinoma treated by radical nephrectomy. Plos one.

[3] Crispen PL (2011). Lymph node dissection at the time of radical nephrectomy for high-risk clear cell renal cell carcinoma: indications and recommendations for surgical templates. Eur Urol.

[4] Ljungberg B (2015). EAU guidelines on renal cell carcinoma: 2014 update. Eur Urol.

[5] MacLennan S (2012). Systematic review of oncological outcomes following surgical management of localised renal cancer. Eur Urol.

[6] Zhang Z (2016). The Difference in Prognosis between Renal Sinus Fat and Perinephric Fat Invasion for pT3a Renal Cell Carcinoma: A Meta-Analysis. PLoS One.

[7] Kim SP (2011). Independent validation of the 2010 American Joint Committee on Cancer TNM classification for renal cell carcinoma: results from a large, single institution cohort. J Urol.

[8] Delahunt B (2009). Advances and controversies in grading and staging of renal cell carcinoma. Mod Pathol.

[9] Brookman-May SD (2015). Evaluation of the prognostic significance of perirenal fat invasion and tumor size in patients with pT1-pT3a localized renal cell carcinoma in a comprehensive multicenter study of the CORONA project. Can we improve prognostic discrimination for patients with stage pT3a tumors?. Eur Urol.

[10] da Costa, W. H. *et al*. Impact of renal vein invasion and fat invasion in pT3a renal cell carcinoma. *PLoS One* **109**, 544–548, 10.1371/journal.pone.0173953 10.1111/j.1464-410X.2011.10366.x (2012). 10.1111/j.1464-410X.2011.10366.x 21711437

[11] Baccos A (2013). Differing risk of cancer death among patients with pathologic T3a renal cell carcinoma: identification of risk categories according to fat infiltration and renal vein thrombosis. Clin Genitourin Cancer.

[12] Pouliot, F., Shuch, B., Larochelle, J. C., Pantuck, A. & Belldegrun, A. S. Contemporary management of renal tumors with venous tumor thrombus. *J Urol* **184**, 833–841; quiz 1235, 10.1016/j.juro.2010.04.071 (2010). 10.1016/j.juro.2010.04.071 20643450

[13] Edge SB, Compton CC (2010). The American Joint Committee on Cancer: the 7th edition of the AJCC cancer staging manual and the future of TNM. Ann Surg Oncol.

[14] Fuhrman SA, Lasky LC, Limas C (1982). Prognostic significance of morphologic parameters in renal cell carcinoma. Am J Surg Pathol.

[15] Lopez-Beltran A, Scarpelli M, Montironi R, Kirkali Z (2006). 2004 WHO classification of the renal tumors of the adults. Eur Urol.

[16] Park M (2017). Prognostic heterogeneity in T3aN0M0 renal cell carcinoma according to the site of invasion. Urol Oncol.

[17] Novara G (2010). Validation of the 2009 TNM version in a large multi-institutional cohort of patients treated for renal cell carcinoma: are further improvements needed?. Eur Urol.

[18] Margulis V (2007). Redefining pT3 renal cell carcinoma in the modern era: a proposal for a revision of the current TNM primary tumor classification system. Cancer.

[19] Abel EJ, Margulis V, Bauman TM (2016). Risk factors for recurrence after surgery in non-metastatic RCC with thrombus: a contemporary multicentre analysis. BJU int.

[20] Jung SJ, Ro JY, Truong LD, Ayala AG, Shen SS (2008). Reappraisal of T3N0/NxM0 renal cell carcinoma: significance of extent of fat invasion, renal vein invasion, and adrenal invasion. Hum Pathol.

[21] Oh JJ (2014). Partial nephrectomy versus radical nephrectomy for non-metastatic pathological T3a renal cell carcinoma: a multi-institutional comparative analysis. Int J Urol.

[22] Khor LY (2016). Tumor Necrosis Adds Prognostically Significant Information to Grade in Clear Cell Renal Cell Carcinoma: A Study of 842 Consecutive Cases From a Single Institution. Am J Surg Pathol..

[23] Pichler M (2012). Trends of stage, grade, histology and tumour necrosis in renal cell carcinoma in a European centre surgical series from 1984 to 2010. J Clin Pathol..

[24] Mouracade, P. *et al*. Perinephric and Sinus Fat Invasion in Stage pT3a Tumors Managed by Partial Nephrectomy. *Clin Genitourin Cancer*, 10.1016/j.clgc.2017.07.019 (2017). 10.1016/j.clgc.2017.07.019 28818550

[25] Poon SA, Gonzalez JR, Benson MC, McKiernan JM (2009). Invasion of renal sinus fat is not an independent predictor of survival in pT3a renal cell carcinoma. BJU Int.

